# Chemokines as Drivers of the Autoimmune Destruction in Type 1 Diabetes: Opportunity for Therapeutic Intervention in Consideration of an Optimal Treatment Schedule

**DOI:** 10.3389/fendo.2020.591083

**Published:** 2020-10-19

**Authors:** Urs Christen, Ruta Kimmel

**Affiliations:** Pharmazentrum Frankfurt/Zentrum für Arzneimittelforschung, Entwicklung und Sicherheit (ZAFES), Goethe University Hospital, Frankfurt am Main, Germany

**Keywords:** CD3, CXCR3, CXCL10, combination therapy, migration, insulitis

## Abstract

Type 1 diabetes (T1D) is mainly precipitated by the destruction of insulin-producing β-cells in the pancreatic islets of Langerhans by autoaggressive T cells. The etiology of the disease is still not clear, but besides genetic predisposition the exposure to environmental triggers seems to play a major role. Virus infection of islets has been demonstrated in biopsies of T1D patients, but there is still no firm proof that such an infection indeed results in islet-specific autoimmunity. However, virus infection results in a local inflammation with expression of inflammatory factors, such as cytokines and chemokines that attract and activate immune cells, including potential autoreactive T cells. Many chemokines have been found to be elevated in the serum and expressed by islet cells of T1D patients. In mouse models, it has been demonstrated that β-cells express chemokines involved in the initial recruitment of immune cells to the islets. The bulk load of chemokines is however released by the infiltrating immune cells that also express multiple chemokine receptors. The result is a mutual attraction of antigen-presenting cells and effector immune cells in the local islet microenvironment. Although there is a considerable redundancy within the chemokine ligand-receptor network, a few chemokines, such as CXCL10, seem to play a key role in the T1D pathogenesis. Studies with neutralizing antibodies and investigations in chemokine-deficient mice demonstrated that interfering with certain chemokine ligand-receptor axes might also ameliorate human T1D. However, one important aspect of such a treatment is the time of administration. Blockade of the recruitment of immune cells to the site of autoimmune destruction might not be effective when the disease process is already ongoing. By that time, autoaggressive cells have already arrived in the islet microenvironment and a blockade of migration might even hold them in place leading to accelerated destruction. Thus, an anti-chemokine therapy makes most sense in situations where the cells have not yet migrated to the islets. Such situations include treatment of patients at risk already carrying islet-antigen autoantibodies but are not yet diabetic, islet transplantation recipients, and patients that have undergone a T cell reset as occurring after anti-CD3 antibody treatment.

## Type 1 Diabetes

It has become clear in the last decades that the predominant destructive force responsible for β-cell death in type 1 diabetes (T1D) are autoaggressive CD8 T cells. Although there is still debate on how the autoimmune response against islet autoantigens is initiated, it seems clear that local inflammation in the islets participates in drawing a broad variety of leukocytes to the islet microenvironment (1). Of course, virus infection has been associated with the etiology of T1D and there is ample evidence to support this hypothesis (2). For example, enterovirus proteins and RNA have been found in islets of T1D patients (3). A large meta-analysis confirmed a significant clinical association between enterovirus infection and T1D (4). However, there is yet no firm, causative proof that would directly demonstrate that virus infection results in immunopathogenic events that would result in the destruction of β-cells and the development of T1D. One problem is the temporal relation between infection and disease onset. Environmental triggers such as virus infection might have occurred long before clinical diagnosis. Further, it is also feasible that more than one triggering event might be required to finally precipitate the disease (5). Thereby, enterovirus infection might as well accelerate a pre-existing autoimmune condition rather than initiate it. Further investigations with more pancreas material, as available from the network for Pancreatic Organ Donors with Diabetes (nPOD), will hopefully shed some more light on the role of virus infection in the etiology of T1D in the future.

For many autoimmune diseases, including T1D, a mechanism termed “molecular mimicry” has been suggested to play a critical role. Molecular mimicry describes a sequential and/or conformational similarity between components of an invading pathogen and the host (6). Cross-reactive antibodies and/or T cells that have been generated during the anti-pathogen defense would thereby also target the similar self-structures of the host and may cause autoimmune damage resulting in clinical disease. Thereby, depending on the strength of the immune tolerance, molecular similarity between pathogen and natural occurring structures of the host is more likely to break tolerance than molecular identity. Many host proteins are expressed in the thymus and central tolerance established to identical molecules might be too strong to be broken. Indeed, in a mouse model for autoimmune hepatitis (7), an infection with a triggering antigen that is similar but not identical to the host target autoantigen was more effective in breaking tolerance and inducing disease as infection of mice that also carry the triggering antigen as a transgenic self-component (8). Interestingly, the immune response to the triggering antigen was focused on epitopes that share an intermediate homology to the host autoantigen, whereas no reactivity was found to regions with high or low homology (8).

The RIP-LCMV-GP mouse model for rapid-onset T1D is based on the concept of molecular mimicry. Such mice express the glycoprotein (GP) of the lymphocytic choriomeningitis virus (LCMV) controlled by the rat insulin promoter (RIP) specifically in the β-cells of the islets of Langerhans in the pancreas (9, 10). T1D is initiated by infecting the mice with LCMV resulting in overt T1D within 10–14 days. LCMV-GP is not expressed in the thymus of RIP-LCMV-GP mice. Thus, although the viral GP is identical with the transgenic GP in the β-cells, LCMV-GP specific T cells are generated that effectively eliminate the virus and attack the β-cells in an autoaggressive manner (10, 11). In contrast to spontaneous models, like the non-obese diabetic (NOD) mouse (12), the RIP-LCMV-GP model allows for a detailed analysis of pathogenic events at defined times after disease initiation, starting from the pathogen-induced acute damage and inflammation to the events driving insulitis and finally the destruction of the β-cells. Thus, besides the valuable information obtained from serum samples as well as pancreas sections of patients with T1D, many insights on crucial inflammatory factors involved in the recruitment of autoaggressive lymphocytes to the islets of Langerhans have been gained from inducible animal models.

## Chemokines as Inflammatory Mediators

Cellular infiltrations into inflamed tissues as occurring in acute or chronic infections as well as autoimmune diseases are orchestrated by chemokines. These chemoattractant inflammatory mediators are mainly released by the local endothelium and by invading immune cells. There is a large, partially redundant, network of chemokines all of which attract a distinct set of migratory leukocytes expressing the corresponding chemokine receptors. Chemokines are classified according to the arrangement of their cysteins in the N-terminal region into the groups CC, CXC, CX_3_C, and XC (13, 14). Specific patterns of chemokines are often found in the serum of patients with autoimmune diseases, but also in those with acute or chronic inflammatory diseases. In order to lure leukocytes to the site of inflammation, many chemokines form a gradient along the endothelial layer by binding to glycosaminoglycans, like heparan sulfate (15). In response to the chemokine gradient migratory leukocytes start rolling along the endothelial layer with the help of selectins that interact with low-affinity (16). In a second step, surface receptors of the integrin family e.g. lymphocyte function-associated antigen 1 (LFA-1) and of the immunoglobulin superfamily of adhesion molecules such as platelet endothelial cell adhesion molecule (PECAM-1) get upregulated and activated, allowing for firm adhesion and finally transmigration of leukocytes through the endothelial cell layer (17). For more detailed information about leukocyte extravasation and the role of adhesion molecules during trafficking to the islets of Langerhans I suggest a recent review by Sandor et al. (18). In general, chemokines are released by local endothelial cells as well as by leukocytes in response to inflammatory factors, including cytokines, such as interferons, interleukins and TNFα. Thereby specific patterns of chemokines seem to emerge locally depending on the corresponding inflammatory disease. Even though glycosaminoglycan-bound chemokines are forming a concentration gradient surrounding the inflamed tissue, many can also be detected in the serum of patients. Due to their partial redundancy and often widespread appearance chemokines are not considered *bona fide* biomarkers for certain diseases. Nevertheless, elevated chemokine serum levels are often associated with autoimmune diseases, including rheumatoid arthritis, multiple sclerosis, or T1D and, together with adhesion molecules, chemokine ligands and their receptors have been and still are considered major drug targets for novel anti-inflammatory therapies (19–21).

## Role of Chemokines in Type 1 Diabetes

The term “insulitis” refers to the local inflammatory milieu with cellular infiltrations and the release of inflammatory factors including chemokines in and around the islets of Langerhans. There are considerable differences in the degree and composition of insulitis between T1D patients and animal models (22). In recent-onset T1D up to one year after diagnosis children (0–14 years) and young adults (14–39 years) still bear 38 and 56% functional islets, respectively. Thereafter, the fraction of functional islets declines to approximately 13% (22). In NOD mice the majority of females develop spontaneous T1D characterized by four stages: Early infiltration at week 4–7 of age, increased insulitis and activation of infiltrating cells at week 8–11 of age, cytotoxicity development with beginning destruction of β-cells at week 12–18 of age, and finally clinical T1D at an age of more than 18 weeks (22). However, the onset of clinical T1D for an individual female NOD mouse can range from 15 to >30 weeks of age. NOD mice display a massive insulitis that is at least one order of magnitude higher than in T1D patients and, in contrast to T1D patients, over time insulitis affects almost all islets in NOD mice. Interestingly, at early stages NOD mice show peri-insulitis, which despite the presence of large clusters of infiltrating cells that show growing similarities with tertiary lymphoid structures (TLO), remains in a surrounding position outside a barrier composed of peri-islet Schwann cells (23) and a peri-islet basement membrane (24). In the fast-onset RIP-LCMV-GP model the events leading to the destruction of the β-cells are more coordinated between individual mice. Cellular infiltration into the islets starts already after about 3 days after LCMV-infection, when neutrophils, macrophages, and dendritic cells enter. By day 7, the first CD4 and CD8 T cells appear and their numbers further increase through days 10 and 14. In contrast to the NOD model, insulitis in the RIP-LCMV-GP model has no clear peri-insulitis stage and cells infiltrate in between β-cells even at an early stage. By days 14–28 post-infection TLO-like clusters of infiltrates are apparent in and around many islets. However, due to the rapid destruction of β-cells and the decline of islet mass after day 28 these clusters get smaller in size and often only islet scars are remaining (25, 26). Overall, in both induced and spontaneous mouse models, insulitis is far more pronounced and the destruction of β-cells occurs faster than in T1D patients. However, the composition of islet-infiltrating cells is similar in T1D patients and NOD as well as RIP-LCMV mice. In particular, insulitis is dominated by T cells that include both islet-antigen specific and non-specific CD8 T cells (27–31). A recent study in human pancreas sections by Bender et al. demonstrated that islet antigen-specific CD8 T cells are found in the islet microenvironment as well as in remote regions of the exocrine pancreas (32). Interestingly, such preproinsulin-specific CD8 T cells are already abundant in the exocrine pancreas of non-diabetic donors. However, during T1D they migrate to and accumulate around and in the islets (32). These data suggest that certain triggering factors, such as local MHC class I upregulation and islet-specific chemokine production, might activate islet antigen-specific CD8 T cells in the exocrine pancreas and guide them to the islets of Langerhans.

Due to the limited access to human pancreas material, the bulk part of collected information about inflammatory factors, such as chemokines, that might be involved in driving the progressing human insulitis is derived from serum assessments. Thus, many chemokines, in particular those associated with a type 1 (Th1/Tc1-associated) T cell response, like CXCL9 and CXCL10, have been found to be elevated in the serum of T1D patients in comparison to healthy donors. For example, elevated serum levels of the type 1 chemokine CXCL10, but not the type 2 (Th2/Tc2-associated) chemokine CCL2, have been found in children with T1D (33). Another study with individuals at high risk for T1D (i.e. 1st degree relatives with multiple autoantibodies) demonstrated increased CCL3 and CCL4 levels, but no change in CXCL10 (34). The problem with all these studies is that on the one hand only small numbers of patients have been analyzed and on the other hand the individual cohorts were in different stages of the disease, ranging from individuals at risk to patients with long established T1D. Anyhow, even if there is no large meta study that would integrate the observations made, the type 1 T cell chemokine CXCL10 seems to be one of the most critical inflammatory mediators that has been associated with the pathogenesis of T1D.

Besides the analysis of serum/plasma samples, additional information has been obtained from isolated islets. The expression of chemokine ligands CCL5, CCL8, CCL22, CXCL9, CXCL10, and CX_3_CL1 has been found in purified human and mouse islets after stimulation with pro-inflammatory cytokines, such as IFNγ and TNFα (35). Further, a transcriptome analysis from islets isolated from NOD and NOD.Rag1-/- control mice at different weeks of age revealed an NOD-specific upregulation of several chemokine ligands (CCL2, CCL4, CCL5, CCL19, CCL22, CXCL9, CXCL10, CXCL11, CXCL13, and XCL1) as well as the presence of chemokine receptors, including CCR2, CXCR4, and CXCR6 on all major leukocyte populations (30). However, there are two problems that arise when working with isolated islets: First, the isolation process itself is often inducing the release of chemokines and other inflammatory factors (36–38) and second, peri-insulitis leukocytes that are mostly only loosely attached to the islets are often lost during the isolation process. Thus, when analyzing the local inflammatory milieu, the expression of chemokine receptors as present on the infiltrating leukocytes might be underestimated. An alternative source for gene expression profiling would be laser-capture micro-dissected islets. Thereby, RNA can be isolated from the entire islet microenvironment, selected regions containing exclusively intact β-cells or infiltrating cells, or even from single β-cells (39). Indeed, laser-capture micro-dissection has been performed in pancreas section of healthy controls, patients with T1D, as well as of several mouse and rat models (37, 40, 41). Although some of these studies have identified proteins involved in immune cell migration to be upregulated in T1D (42), the focus of these studies was often on other topics and therefore a clear conclusion on the role of chemokines is still missing. One reason for this lack of solid data might be the broad time frame in which T1D is manifesting in patients as well as in spontaneous models, such as the NOD mouse, that prevents a detailed analysis of inflammatory events playing a role in the various phases of T1D immunopathology. In contrast, inducible models with a defined starting point and a tightly synchronized schedule of pathogenic events allow a detailed mapping of the expression pattern over time with only little inter-individual variations. Several studies in the inducible RIP-LCMV model using RNA from whole pancreas or from isolated islets as well as performing immunohistochemistry of pancreas sections at several defined times after initiation of T1D by LCMV infection demonstrates that during the immunopathogenesis of T1D a broad variety of chemokines are released with distinct kinetic patterns (26, 35, 43). As in the study by Carrero et al. with NOD-islets (30), an increase in chemokine receptors occurs after the expression of chemokine ligands has been upregulated, indicating the presence of chemokine receptors on infiltrating cells. A selection of studies that identified chemokines in patients with T1D and/or experimental animal models for T1D is displayed in **Table 1**.

**Table 1:** Chemokine expression in T1D patients and experimental animals.

Species	Location	Chemokine	Reference
*Human*			
Children/adults with T1D	Serum	**CXCL10**	(44, 45)
Individuals at high risk for T1D	Serum	CCL3, CCL4	(34)
Newly diagnosed T1D patients	Serum	**CXCL10**	(33)
Recent onset T1D patients	Islets (IHC)	**CXCL10**	(46)
Recent onset T1D patients	β-cells (IHC)	**CXCL10**	(47)
T1D patients	Stimulated isolated islets (mRNA)	CCL5, CCL8, CCL22, CXCL9, **CXCL10**, CX_3_CL1	(35)
T1D patients	Islets (IHC)	CCL5, CCL8, CXCL9, **CXCL10** >> CX_3_CL1	(35)
Newly diagnosed T1D patients	Serum	CCL2, CXCL8, CXCL9, **CXCL10**	(48)
*Spontaneous T1D models*			
NOD mice	Specific BDC T cells (mRNA)	CCL2, CCL3, CCL4, XCL1 >> CCL5, **CXCL10**	(49)
NOD mice	β-cells (IHC)	**CXCL10**	(35)
NOD mice	Islets (mRNA transcriptome),	CCL2, CCL4, CCL5, CCL19, CCL22	
		CXCL9, **CXCL10**, CXCL11, CXCL13, XCL1	(30)
BB rat	Islets (mRNA transcriptome),	CCL2, CCL3, CCL19, CCL20, CCL21	
		CXCL1, **CXCL10**	(50)
*Inducible T1D models*			
RIP-LCMV mice	Pancreas (mRNA)	CCL5, CXCL9, **CXCL10** >> CCL11, XCL1	(26)
RIP-LCMV mice	Islets (mRNA)	CXCL9, **CXCL10** >> CCL2, CCL5, CXCL2	(43)
RIP-LCMV mice	Islets (IHC)	α-cells: CXCL9; β-cells: **CXCL10**	(43)
Prediabetic RIP-LCMV mice	Islets (IHC)	α-cells: CX3CL1; β-cells: CCL8, **CXCL10**	(35)
RIP-LCMV mice (islet transplantation)	Islets (IHC)	**CXCL10**	(38)
STZ-islet transplantation model	Serum	CCL2, CCL22, **CXCL10**	(51)

This table lists a selection of publications reporting chemokine expression in patients with T1D and/or experimental animal models for T1D. Note that most studies have identified CXCL10 as one of the most apparent chemokines expressed.

BB rat, Biobreed rat; IHC, Immunohistochemistry; STZ, Streptozotocin.

## The CXCL10/CXCR3 Chemokine Axis in Type 1 Diabetes

The chemokine CXCL10 has been identified quite a while ago to have a dominant role in the attraction of effector T cells bearing the corresponding receptor CXCR3. It has been reported that CXCL10 is elevated in the serum of long-standing (44) as well as newly diagnosed (45) T1D patients. However, the serum concentration was lower in patients with long-standing T1D. Interestingly, the mean serum CXCL10 level was also found to be elevated in patients at high risk for developing T1D who are carrying antibodies against islet autoantigens indicating that CXCL10 is released during the initial β-cell destructive process (44, 45). Although the observed elevation has not been confirmed in two other similar studies (34, 52), it is important to note that in the study by Rotondi et al. the range of serum CXCL10 concentrations was much broader in newly-diagnosed T1D patients than in healthy controls and the highest concentration found in patients was more than five times higher than in controls (52). A more detailed study has been performed by Antonelli et al. who analyzed CXCL10 serum levels in 96 newly diagnosed children with T1D at the time of diagnosis and at a median follow-up time of 16 months (33). Indeed, almost half of the newly diagnosed children had elevated CXCL10 levels compared to a healthy control group. In addition, the study confirmed the earlier finding that the CXCL10 serum levels decline over time but are still elevated even 16 months after diagnosis when compared to levels found in control individuals (33).

It has been found that CXCL10 as well as its main receptor CXCR3 are expressed directly in the islet microenvironment of T1D patients (35, 46, 47). Roep et al. stained pancreas sections from three new-onset T1D patients and found CXCL10 expression by β-cells and CXCR3 expression by infiltrating lymphocytes (46). This observation was confirmed by Uno et al. who performed double-immunofluorescence staining of pancreas sections of five recent-onset T1D patients. They clearly identified β-cells as a main source of CXCL10 and T-cells as the main cell type expressing CXCR3 in the islet microenvironment (47). Further, an extensive study by Sarkar et al. using RNA obtained from isolated human islets of four independent donors identified CXCL10 as the dominant chemokine expressed in islets of T1D patients (35). Since the peak of CXCL10 expression seems to be at the time of diagnosis or maybe even before clinical manifestation in islet autoantibody positive individuals rather than during the chronic phase of established T1D the question for the trigger of CXCL10 release arises. In this context an intriguing study by Tanaka et al. revealed enterovirus capsid protein VP1 expression in pancreata of three patients with fulminant T1D (FT1D) and ketoacidosis together with CXCL10 expression in α- and β-cells as well as strong islet-infiltration by CXCR3-positive T cells (53). The group recently further confirmed these data in a more detailed study using pancreas tissue of three FT1D patients and 17 healthy controls. They found that close association between VP1 and CXCL10 expression is not only detected in islet cells, but also in pancreatic exocrine ductal cells and acinar cells and concluded that enterovirus infection induced CXCL10 expression in both exocrine pancreas and islets (54).

Since access to human pancreas tissue and experimental evaluations of immunopathological mechanism in T1D patients are limited, research has focused on experimental animal models for T1D. Thereby, CXCL10 has also been identified as one of the key chemokines expressed during experimental T1D. In particular, in model systems using virus-infection to induce the autoimmune destruction of β-cells, such as the RIP-LCMV mouse model, CXCL10 was among the first chemokines upregulated in the pancreas upon LCMV-infection (26). However, CXCL10 was also found to be upregulated during the pathogenesis of T1D in NOD mice (49). Stimulation of isolated human, rat, and mouse islets with pro-inflammatory cytokines (TNFα, IFNγ) induced the expression of CXCL10, demonstrating the capacity of the islets themselves to be able to express CXCL10 (55). Indeed, immunohistochemical analysis of mouse pancreas sections has indicated that similar to the findings in patients, CXCL10 is mainly generated by β-cells and CXCR3 is present on infiltrating leukocytes, including CD8 T cells (38, 43, 46).

It has also been shown that T1D is milder in mice lacking CXCL10 or CXCR3 (43, 56). In contrast, T1D was accelerated in mice overexpressing CXCL10 in the β-cells (28). Interestingly, such RIP-LCMV x RIP-CXCL10 double transgenic mice displayed large clusters of infiltrating cells around and inside the islets of Langerhans but did not develop T1D spontaneously. Nevertheless, islet stress seemed to be present since such mice needed much longer to return to normoglycemia after glucose challenge (28). Thus, in these double-transgenic mice CXCL10 recruited leukocytes to the islets, but without activation and further expansion of islet antigen-specific T cells by LCMV-infection, β-cells were not actively destroyed. Collectively, these data suggest that CXCL10 orchestrates the migration of islet antigen-specific as well as non-specific T cells to the site of inflammation. In consequence, when a high concentration of CXCL10 is present at an auxiliary site responsive T cells might be recruited away from the islets. Indeed, when LCMV-infected RIP-LCMV mice receive an additional virus infection that grows to high titers in the lymph nodes causing a high local concentration of CXCL10, islet antigen-specific T cells accumulate in the infected lymph nodes and are driven to apoptosis by hyperactivation, resulting in an abrogation of T1D (57).

From a therapeutic perspective, it is important that a blockade of the CXCL10/CXCR3 axis with a neutralizing antibody reduced the T1D incidence in mice (26, 58). In the RIP-LCMV model the incidence of T1D was found to be reduced by about 70% after administration of an anti-CXCL10 antibody (26). Thereby the reduction of T1D went together with a reduced insulitis, maintained insulin production, and a reduced frequency of islet antigen-specific T cells. Nevertheless, in a follow-up study, it has been shown that there is a certain redundancy in the CXCL10/CXCR3 axis since the reduction of T1D in CXCR3-deficient RIP-LCMV mice and in anti-CXCL10 antibody treated RIP-LCMV mice was not as pronounced as in earlier studies (Coppieters et al., 2013). Importantly, it should be noted that the anti-CXCL10 antibody treatment started before initiation of the disease by LCMV-infection and therefore like using CXCL10-deficient mice the CXCL10 neutralization constituted a preventive rather than a therapeutic intervention. An anti-CXCL10 antibody treatment of already diabetic RIP-LCMV mice starting at day 13 after LCMV-infection resulted only in a slight, non-significant reduction by about 25% (56). One reason for this lack of efficacy is that at the start of the therapy the autoaggressive T cells have already assembled in the islet microenvironment and have started to progressively destroy β-cells. By that time, CXCR3-positive cells might even be trapped in the pancreatic lymph nodes or the TLO-like inflammatory clusters in and around the islets. Thus, the question about the perfect time for treatment arises. Whereas an answer to this question might be found in inducible models, such as the RIP-LCMV model, it is much more of a central problem for a therapy of T1D patients. Insulitis is already detected in new-onset T1D patients and, like in new-onset diabetic mice, CXCL10 neutralization might come too late. Should the hypothesis (2, 59) that one or more pathogens are involved in the initiation and/or propagation of the β-cell destructive process be correct, a CXCL10 neutralization would have to begin immediately after the critical infectious event. Thus, as long as there is no firm proof for (a) particular pathogen(s) to directly induce T1D, such a therapeutic scenario is highly unlikely.

There are however other occasions that would allow for a more reasonable therapeutic intervention. One occasion would be islet or whole pancreas transplantation. Here, the precise time of transplantation is of course known and ideally the new tissue should be devoid of autoaggressive T cells by the time of transplantation. In theory, a neutralization of CXCL10 would therefore not come too late and would reduce the *de novo* infiltration of the transplant. Indeed, the majority of diabetic RIP-LCMV mice transplanted with CXCL10-deficient islets under the kidney capsule did not reject the islets. In addition, neutralization with an anti-CXCL10 antibody significantly delayed the islet rejection in such a setting (38). Similar data have been obtained in mice administered streptozotocin (STZ) to induce T1D (51). The second occasion would be a T cell reset situation as occurring after a partial T cell depletion caused by an anti-CD3 antibody therapy. Thus, chemokine neutralization might be better suited as part of a combination therapy.

## Chemokine Neutralization as Part of a Combination Therapy

Chemokine ligands and especially their receptors have been used as targets for immune intervention for decades (20, 60). However, most of the clinical trials have been terminated after phase II or even earlier indicating an overall lack of efficacy. Maraviroc, which targets CCR5 and is used after infections with human immunodeficiency virus (HIV), is one of the few exceptions (61). Though, interfering with HIV entry is mechanistically completely different from blocking chemokine guided immune cell migration. One of the reasons for the lack of efficacy of drugs interfering with any chemokine axes might be that, as described above for mice with already established T1D, even a treatment of patients just recently diagnosed with any autoimmune-related disease might come too late. A possible solution to this impediment might be to set a fresh starting point for the autoimmune destruction process by temporarily depleting the culprit cells responsible for the destruction. In the case of T1D, the major destructive force are the autoaggressive T cells. T cell depletion/reprogramming has been performed using several variations of anti-CD3 antibody treatment since more than twenty years. Initially, experiments in NOD mice had demonstrated that anti-CD3 antibody administration induces a remission of T1D by re-establishing self-tolerance to islet autoantigens (62, 63). Mechanistically, an anti-CD3 antibody treatment causes on the one hand an inactivation of aggressive T cells and on the other hand results in an expansion of regulatory T cells (64).

The efficacy of anti-CD3 antibodies in T1D, such as teplizumab (hOKT3g1) and otelixizumab (ChAglyCD3), has been evaluated in several clinical trials, including the DEFEND-1 (otelixizumab), Protégé (teplizumab), and AbATE (teplizumab) studies (65–68). In such trails, patients with new-onset and recently diagnosed T1D were infused for a short period of time of only 6 to 14 days with an anti-CD3 antibody. In the Protégé study the patients received a second treatment cycle at week 26 after the start of the first cycle (66, 69). Overall, the therapeutic success lasted for about one to two years. Thereafter, the C-peptide levels and the insulin need approached those values of the placebo treated arm (66, 70, 71). Interestingly, in the AbATE study more than 50% of the patients did not respond to the anti-CD3 antibody treatment, whereas responders showed restored C-peptide levels for up to two years after treatment (71). However, a seven-year follow-up study revealed that even in drug responders the C-peptide levels declined massively in the time between two and seven years post-treatment (72). Thus, in order to uphold the protection for an extended period of time, the anti-CD3 treatment should be repeated or be combined with a secondary treatment. Whereas a repetitive administration of anti-CD3 antibodies has not been considered due to a high probability of severe side effects of the rather unspecific immune suppression, several combination therapies have been evaluated in animal models. They included administration of nasal proinsulin (73), Lactococcus lactis secreting IL-10/proinsulin (74), cyclosporine A and vitamin D3 analog (TX527) (75), anti-CD20 antibody (76), fingolimod (FTY720) (77), or the selective sphinogosine-1 phosphate-1 (S1P1) modulator ponesimod (78), just to name a few. Most of these combination therapies improved the outcome compared to the monotherapies. Similar to the therapies with anti-CD3 antibody/fingolimod (77) and anti-CD3 antibody/ponesimod (78) the combination therapy with anti-CD3 antibody and CXCL10 neutralization targeted the migration of the regenerated T cells into the islets of Langerhans (56).

**Figure 1:**
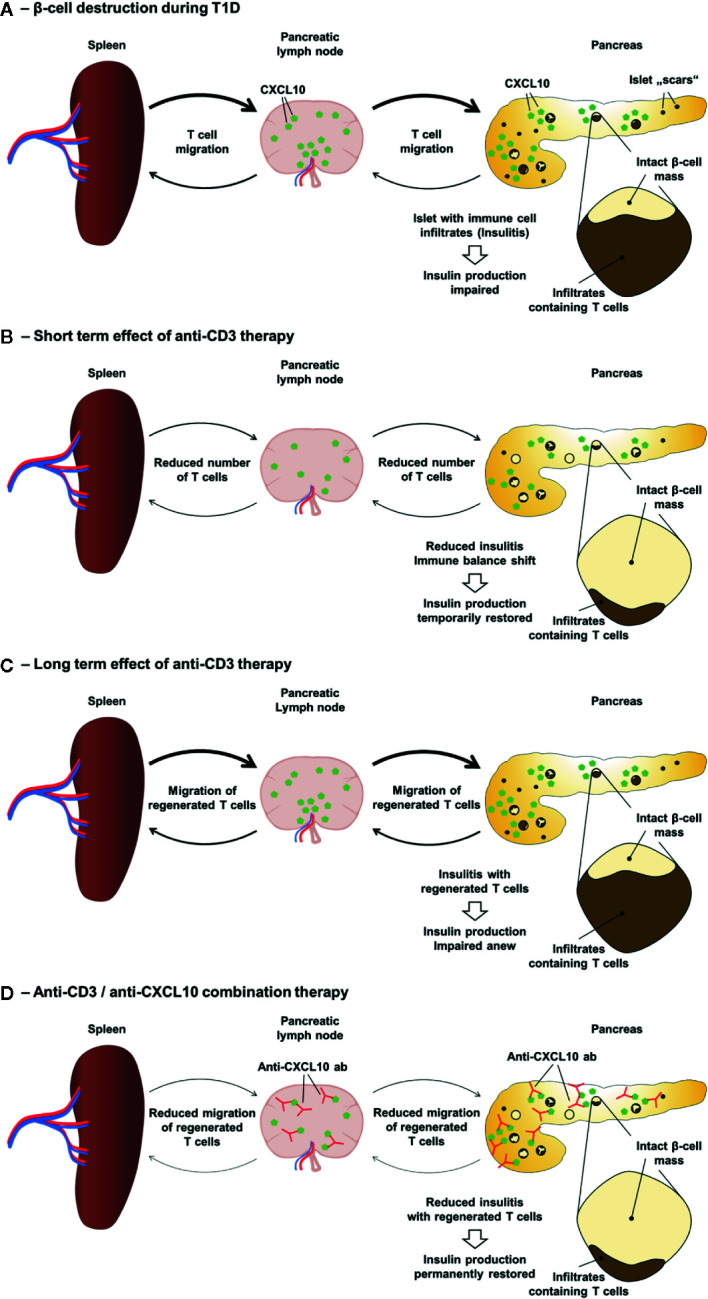
Anti-CD3/anti-CXCL10 combination therapy for type 1 diabetes. **(A)** Local expression of CXCL10 and other chemokines in the islets of Langerhans as well as in the pancreatic lymph nodes drive the migration of leukocytes, including autoaggressive T cells to the islets. Due to β-cell destruction and stress the insulin production is insufficient to control the blood glucose level. **(B)** Anti-CD3 therapy causes a partial depletion of T cells and induces an immune balance shift resulting in a reduced insulitis and a temporarily restored insulin production. **(C)** However, in T1D patients anti-CD3 therapy only lasts for 1–2 years and in diabetic mice only about 30% go into remission. Regenerated T cells migrate to the islets and the self-destructive process start anew resulting in an impaired insulin production. **(D)** Administration of neutralizing anti-CXCL10 antibodies after the anti-CD3 therapy inhibits the migration of regenerated T cells and thereby prevents the re-infiltration of the islets resulting in a permanent T1D remission.

As mentioned above, an anti-CD3 antibody therapy provides a “reset” situation regarding the presence of aggressive T cells in the islets of Langerhans. The subsequent neutralization of the CXCL10/CXCR3 axis might prevent the re-infiltration of the islets by autoaggressive T cells that have regenerated in spleen and lymph nodes (**Figure 1**). Indeed, in both new-onset diabetic RIP-LCMV as well as NOD mice an administration of three daily doses of a non-Fc-binding anti-CD3ϵ F(ab’)2 fragment [clone 145-2C11] (63) followed by a treatment with a neutralizing anti-CXCL10 antibody [clone 1F11] (79) resulted in profound remission of T1D (56). Thereby the combination therapy was superior to the corresponding monotherapies with anti-CD3 antibody or anti-CXCL10 antibody alone. Importantly, the observed remission was long lasting, since none of the cured mice relapsed until the end of the observation period of six months. As to be expected, insulitis was strongly reduced in both models. However, whereas after combination therapy the frequency of islet antigen-specific CD8 T cells was greatly reduced in the pancreas of remitting RIP-LCMV mice, there was no further reduction in NOD mice receiving the combination therapy over those that were administered with anti-CD3 antibody only. In contrast, the frequency of regulatory T cells was further elevated after combination treatment in NOD, but not RIP-LCMV mice. Interestingly, the local ratio of regulatory T cells to islet antigen-specific effector CD8 T cells was strongly enhanced after combination therapy of both RIP-LCMV and NOD mice, indicating a vital shift in the immune balance locally in the islets (56). The crucial role of CXCL10 in the islet re-infiltration process is also underlined by the fact that T1D was completely abolished in CXCL10-deficient RIP-LCMV mice after treatment with anti-CD3 antibody (56). Thus, as outlined in **Figure 1**, a well-timed partial deletion and reprogramming of T cells through short-term administration of anti-CD3 antibodies followed by the inhibition of T cell migration and islet re-infiltration *via* neutralization of the key inflammatory chemokine CXCL10 induces a persistent remission of T1D.

**Figure 2:**
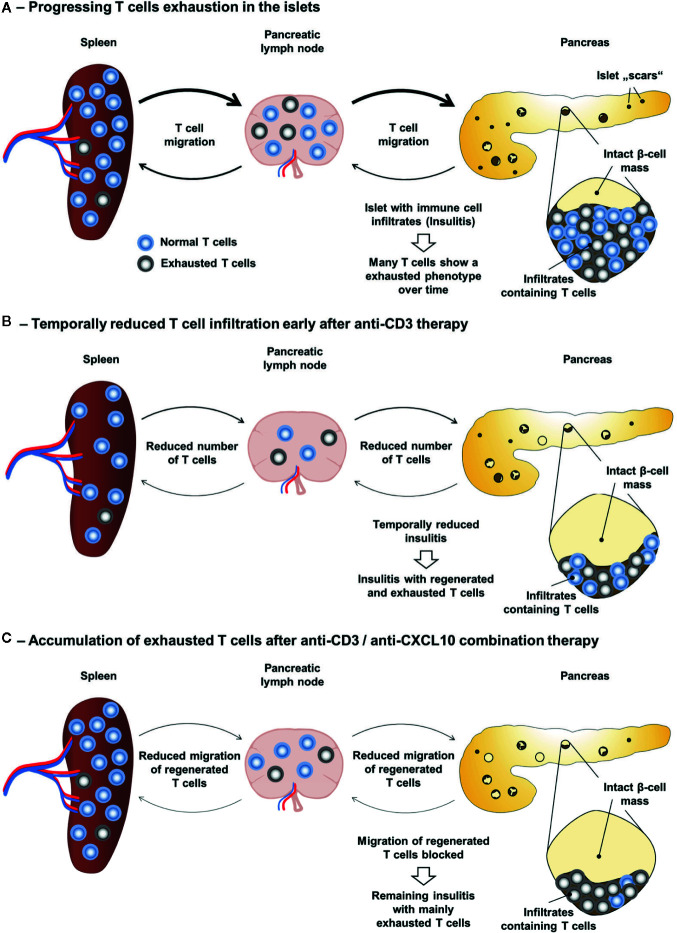
Accumulation of exhausted T cells as possible explanation for the long-lasting effect of the anti-CD3/anti-CXCL10 combination therapy. **(A)** During the progressive destruction of β-cells in the RIP-LCMV-GP model the majority of islet autoantigen (LCMV-GP) specific T cells enter a state of exhaustion. **(B)** Upon anti-CD3 therapy the frequency of T cells is temporarily reduced and the frequency of exhausted T cells is increased. However, upon termination of the anti-CD3 antibody treatment, newly regenerated T cells are not prevented from invading the islet microenvironment. **(C)** In contrast, anti-CD3/anti-CXCL10 combination therapy prevents the migration of newly regenerated, functionally active T cells to the islets and results in an increased frequency of exhausted islet autoantigen-specific T cells in the islet microenvironment.

Persistent LCMV infection is causing a functional exhaustion of specific CD8 T cells (80, 81). Thereby, the expression of inhibitory receptors, such as PD-1 (programmed cell death protein 1), is considered a hallmark of T cell exhaustion (82). Indeed, blockade of PD-1 restores the cytotoxic function of exhausted CD8 T cells and reduces the viral titer (83). It has also been recently shown that the TcR signalling is strongly inhibited in such exhausted T cells (84). Although LCMV infection in the RIP-LCMV model for T1D is not persistent, the chronic transgenic β-cell expression of LCMV-GP seems to result in a similar phenomenon of functional T cell exhaustion. During the destruction of β-cells in the RIP-LCMV model many T cells in the pancreas show an exhausted phenotype (85). Importantly, treatment of T1D patients with teplizumab as conducted in the AbATE trail resulted in an increased frequency of T cells with an exhaustion phenotype (86). A seven-year follow-up study even revealed that T cell exhaustion may serve a biomarker for response (72). Therefore, besides the observed shift in the immune balance towards a more regulatory milieu (56), another possible factor involved in the long-lasting effect of the anti-CD3/anti CXCL10 antibody combination therapy might be a predominance of exhausted T cells in the islet microenvironment. Thereby, the anti-CD3 antibody treatment would cause an accumulation of exhausted T cells and the subsequent blockade of the CXCL10-CXCR3 axis would prevent the re-infiltration of newly regenerated, functionally active T cells (**Figure 2**).

## Summary and Future Perspectives

It seems clear that chemokines are important for the attraction of autoaggressive immune cells, including islet-autoantigen-specific T cells, to the islet of Langerhans. Several chemokines have been identified to be elevated in the serum of T1D patients or expressed by the islet microenvironment of mice. The CXCL10/CXCR3 chemokine axis is of particular interest, since patients with T1D display enhanced serum levels of CXCL10, both CXCL10 as well as CXCR3 have been found in pancreas tissue sections from T1D patients, and neutralization studies in mouse models demonstrated a reduction of T1D incidence and severity. However, most of the studies done in mice are not applicable to the human situation, since the precise moment of disease initiation in humans is not known and treatment might therefore come too late. Even if treatment would start in individuals at risk that have already generated autoantibodies against two or more islet autoantigens a migration blockade might be behind schedule since insulitis has very likely already started. Thus, neutralization of chemokines seems more appropriate in situations with no or only mild insulitis. The first situation would be whole pancreas or islet transplantation. Even though the transplantation process as such is initiating inflammatory responses, the islets at that point of time are largely devoid of insulitis. The second situation is a partial T cell depletion as occurring after short-term anti-CD3 antibody treatment. In patients, a monotherapy with anti-CD3 antibodies has proven to be beneficial, still many patients did not respond and even in responders the remission lasted only for one to two years. In two independent mouse models, a well-timed combination therapy of anti-CD3 antibody administration followed by an additional blockade of the CXCL10-CXCR3 axis prevented the re-infiltration of the islets by regenerating T cells and thereby induced a persistent remission in the majority of treated mice. No severe adverse effects have been reported for patients receiving two cycles of anti-CD3 antibody treatment within 26 weeks (66, 69). Nevertheless, a repetitive treatment with cycles of anti-CD3 antibody infusions over several years or even decades has not yet been considered, due to possible long term adverse effects, such as reduced pathogen defense, viral reactivation, and enhanced risk for tumor development. Due to the partial redundancy of the chemokine ligand-receptor network the blockade of a single chemokine axis, that plays a crucial role in the pathogenesis of a particular autoimmune disease, is likely to have a lower impact on the general immune defense than a partial T cell depletion. Thus, there is still need for alternative combination therapies that abstain from anti-CD3 antibody administration. Ideally such combinations should include targets that are mechanistically involved in separate steps of the immunopathogenesis. Thus, a parallel or sequential neutralization of several chemokine axes involved in the attraction of distinct leukocyte populations such as aggressive T cells and dendritic cells might resolve already existing leukocyte clusters in TLO-like structures around and in the islets of Langerhans without prior anti-CD3 antibody treatment. In any case, to achieve persistent T1D remission, it is important to find novel combination therapies that can be administered in a well-timed regimen in patients with newly diagnosed or even established T1D.

## Author Contributions

UC and RK have been writing the review text and designed the figures. All authors contributed to the article and approved the submitted version.

## Funding

No direct funding has been received for this review article. Discussed data from our research group have been realized through funding to UC by the German Research Foundation (DFG) CH 806/1-1, the Else Kröner-Fresenius Foundation (EKFS), Research Training Group Translational Research Innovation Pharma (TRIP), the Landesoffensive zur Entwicklung wissenschaftlich‐ökonomischer Exzellenz (LOEWE), Center ‘Translationale Medizin und Pharmakologie’ (TMP) TMP-IF-01, and the Hospital of the Goethe University Frankfurt.

## Conflict of Interest

The authors declare that the research was conducted in the absence of any commercial or financial relationships that could be construed as a potential conflict of interest.
